# Management Strategies for Borderline Personality Disorder and Bipolar Disorder Comorbidities in Adults with ADHD: A Narrative Review

**DOI:** 10.3390/brainsci13111517

**Published:** 2023-10-26

**Authors:** Luke MacDonald, Joseph Sadek

**Affiliations:** 1 Faculty of Medicine, Dalhousie University, Halifax, NS B3H 4R2, Canada; lukemacdonald@dal.ca; 2 Department of Psychiatry, Dalhousie University, Halifax, NS B3H 4R2, Canada

**Keywords:** ADHD, borderline personality disorder, bipolar disorder, management, dual diagnosis, psychostimulants, psychotherapy

## Abstract

This narrative review examines two of the common comorbidities of attention-deficit/hyperactivity disorder, bipolar disorder (BD), and borderline personality disorder (BPD), which each share several common features with ADHD that can make assessment and diagnosis challenging. The review highlights some of the key symptomatic differences between adult ADHD and these disorders, allowing for more careful consideration before establishing a formal diagnosis. When the disorders are found to be comorbid, further complications may arise; thus, the review will also help to provide evidence-based treatment recommendations as well as suggestions on how to minimize adverse events. Incorporating evidence from systematic reviews, journal articles, randomized controlled trials, and case reports, this review highlights that the diagnosis of ADHD and some of its common comorbidities is challenging and requires full, in-depth assessment and management. The management strategies of these comorbidities will also be addressed, with emphasis on achieving mood stabilization for BD prior to initiating appropriate ADHD pharmacotherapy. Medications, specifically mood stabilizers, antipsychotics, and antidepressants, are fundamental in treating symptoms seen in BD and some cases of BPD, alongside psychotherapy and lifestyle modifications when appropriate. The review highlights the effectiveness of specific medications, including psychostimulants, atomoxetine, and bupropion, as add-on therapies to mood-stabilizing treatments for addressing ADHD symptoms in patients with these comorbidities. Despite limited research, the review will address various pharmacological and psychotherapeutic approaches for managing comorbid ADHD and BPD, emphasizing the need for further investigations to better understand the unique needs of this patient population.

## 1. Introduction

Throughout history and now into modern practice, ADHD has been regarded as a neurodevelopmental disorder in children—often slated to self-resolve during adolescence and young adulthood [1]. In recent years, however, ADHD has evolved to become more intricately understood, and it has been determined that roughly 65% of cases involve the persistence of symptoms into adulthood [2]. While there have been changes in the DSM-IV and DSM-V to reflect the persistence and presentations of ADHD in adulthood, adult patients with this disorder continue to face barriers to getting sufficient support [3]. ADHD is strongly associated with psychiatric comorbidities; it is reported that roughly 80% of adults with ADHD have at least one coexisting psychiatric disorder, with rates of comorbid bipolar disorder (BD) between 5 and 47%, rates of depression between 9 and 16%, and anxiety rates approaching 50% in the adult ADHD population [4]. There is also a notable increased risk of developing psychiatric comorbidities in individuals who have ADHD, including anxiety disorders, substance use disorders, depression, and bipolar disorder [5]. The most common comorbidities in ADHD include anxiety disorders, substance use disorder (SUD), and personality disorders, with SUD being twice as common in the ADHD population than the general population—with a predilection towards nicotine, cannabis, and cocaine [4]. Personality disorders are also found in roughly half of the adult ADHD population, and researchers identified cluster B and C disorders to be among the most common [4]. This same pattern is also observed in patients with bipolar disorder [6]. The presence of comorbidities may result in delayed recognition and treatment of ADHD.

While comorbidities vary across the psychodiagnostic spectrum, literature gaps present themselves when identifying the relationship of ADHD to personality disorders and bipolar disorder in particular—two very different but burdensome disorders that are often observed in patients with ADHD. Reports suggest that personality disorders are present in more than 50% of adults with ADHD—with particular attention focused on cluster B disorders like borderline personality disorder (BPD) [7]. This is an important concept for the management of both issues because their concurrence with one another is linked to more severe impairment and poorer response to ADHD pharmacotherapy [4].

Both bipolar I disorder and bipolar II disorder are found at higher rates among adult patients with ADHD [8]. A recent meta-analysis demonstrated that roughly 1 in 13 adults with ADHD has an additional diagnosis of bipolar disorder, while 1 in 6 bipolar patients are also living with ADHD; interestingly, it appears that there is no disparity between the rates of bipolar I disorder vs. bipolar II disorder in the adult ADHD population [9]. There are clinical characteristics that overlap in patients presenting with either bipolar disorder or ADHD, so it can pose a unique challenge to clinicians who may struggle with the identification of certain psychopathologies. Studies suggest that comorbid ADHD and bipolar disorder are linked to worsened course of illness and more frequent episodes of mania and depression [4]. With a modernized perspective on the importance of correctly diagnosing and managing ADHD and comorbid psychiatric disorders in adulthood, it is crucial that clinicians gain a solid understanding of how the presence of other mental health concerns in patients with ADHD may warrant more in-depth analysis and treatment modifications to help deliver optimized care.

## 2. Methodology

### 2.1. Study Design

This narrative review employs a comprehensive approach to explore the management strategies for comorbid ADHD with BD and BPD in adults. Evidence from a range of peer-reviewed research articles, clinical guidelines, case reports, and expert opinions will provide a comprehensive overview of the topic in question.

### 2.2. Literature Search

A search of academic databases, including PubMed, Embase, PsychINFO, and Google Scholar, was conducted to identify relevant articles published up to the knowledge cutoff date of July 2023. The search terms used included various combinations of “ADHD”, “bipolar disorder, “borderline personality disorder”, “comorbidity”, “management”, and “treatment strategies”.

### 2.3. Inclusion Criteria

1. Articles published in peer-reviewed journals between 1999 and 2023 in the English language.
2. Studies addressing the adult population (both males and females) with ADHD and comorbid disorders and patients treated either with pharmacotherapy, psychotherapy, or both.
3. Randomized control trials, systematic reviews, metanalysis, and observational studies were included.

### 2.4. Exclusion Criteria

1. Articles that primarily focused on pediatric populations since borderline personality cannot be diagnosed prior to age 18.
2. Articles written without relevance to ADHD, BD, or BPD. Secondary reports were excluded.
3. Articles published prior to 1999.
4. Qualitative studies of ADHD and comorbid disorders

### 2.5. Data Extraction and Synthesis

The selected articles were reviewed independently and analyzed for relevance to the topic in question. Data extracted included information on treatment goals, medication regimens, psychotherapeutic approaches, and key points on diagnostic criteria and treatment in adults with ADHD and comorbid BD or BPD. The data collected were then synthesized to provide a coherent narrative review that addresses the research objectives. Emphasis was placed on the most relevant and logical treatment approaches for patients with these types of presentations.

## 3. Diagnostic Considerations

### 3.1. ADHD and Bipolar Disorder

Describing the diagnostic overlap of ADHD and bipolar disorder poses a challenge, specifically because of the cyclic nature of bipolar disorder symptoms. While there may be features commonly seen in manic or depressive episodes that are consistent with an ADHD presentation, it is important to take note of the transient nature of bipolar mania. The symptoms that tend to overlap between the two conditions are those that tend to present in a more subtle fashion—including persistent low self-esteem, issues with performance in school or work, and the presence of rapidly flowing thoughts that may be described as a ‘restless mind’ [10]. These are features that are often seen in patients with ADHD *and* patients with BD and, therefore, must be considered when identifying a diagnosis of either disorder in a presenting patient.

Patients with BD who are experiencing a manic episode often present with patterns that involve excessive talkativeness, distractibility, and restlessness—all of these clinical features can also be observed in patients with ADHD [11]. There are aspects seen in mania, however, that are not characteristic of a typical ADHD patient—these include elevated mood, decreased need for sleep, and an inflated sense of self-importance or grandiosity [4]. In severe manic episodes, patients may experience psychotic symptoms like hallucinations and delusions. The main features of ADHD are inattention, impulsivity, and hyperactivity—with inattention and impulsivity predominating in adulthood [1]. These are also symptoms that can be observed in bipolar disorder, particularly in the manic and hypomanic states. However, the features of these symptoms can be differentiated with some key characteristics; inattention in ADHD is characterized by being easily distracted and experiencing wandering thoughts, while in manic and hypomanic episodes, it is better described as altered clarity of thought, flight of ideas, and associated thought acceleration [12]. Meanwhile, impulsivity in ADHD is often observed as an inability to stop, relax, and wait—leading to a presentation that is consistent with intrusiveness and impatience. There are two domains of impulsivity in the ADHD literature, described as cognitive impulsivity and motor impulsivity [13]. Cognitive impulsivity involves impulsive decision-making and is typically associated with making poorly thought out decisions, while motor impulsivity is described as behaviors like restlessness and interrupting (on behalf of the International Multicentre Persistent ADHD CollaboraTion [14]. Impulsivity in bipolar disorder, on the other hand, is a feature of manic episodes, which are typically fueled by elevated mood, racing thoughts, and increased energy [11]. Behaviors that are often seen as a result of the manic state may include uncontrolled spending, promiscuity, and suicide attempts. It may be linked to the element of grandiosity and is associated with underestimation of consequences [12].

It has been reported that the 12-month prevalence of bipolar disorder in the ADHD population is up to 7.4× higher than that of the general population [15]. Studies have also demonstrated that patients with comorbid ADHD and BD tend to experience more severe mood disorder symptoms and more frequent episodes of bipolar-associated mood states [16]. The data presented from the Systematic Treatment Enhancement Program for Bipolar Disorder (STEP-BD) study demonstrated that only 9% of patients with ADHD-BD were properly diagnosed and treated for their ADHD [17]. Given their commonality and the apparent difficulty in the recognition and appropriate management of these patients, it will be important for mental health clinicians to correctly identify and help patients achieve optimal management of these two complex and debilitating diagnoses.

### 3.2. What Are the Main Distinguishing Features between ADHD and Bipolar Disorder?

There are also some features of BD mood episodes that are not seen in ADHD, which may help to distinguish between a bipolar manic episode and ADHD-related hyperactivity (see Table 1). For instance, manic episodes may often involve changes in sexual behavior, typically in the form of increased sex drive and engaging in riskier sexual practices [18]. Conversely, changes in sexual activity are not observed in patients with an isolated ADHD diagnosis, largely because impulsivity in ADHD relates more to cognitive control and executive functioning rather than elevated mood [12].

Grandiosity, or an inflated sense of self-esteem, is a prominent feature of bipolar mania or hypomania; these individuals frequently present with inflated or unrealistic perceptions about their own importance or abilities [11]. These characteristics are typically not observed in patients with ADHD, who are not as likely to experience the euphoria, mood disturbances, and distorted sense of self that are seen in patients with BD [11]. Similarly, episodes of psychosis (delusions or hallucinations that impact the patient’s perception of reality), which can occasionally occur in bipolar mania, would not be a typical feature seen in adults with ADHD [19].

Sleep disturbances are a common feature of several psychiatric disorders, and both ADHD and BD are known to impact sleep—however, they do so in different ways, something that skilled diagnosticians are aware of. While ADHD is linked to sleep disturbances, it is not known to decrease the need for sleep, which is a feature seen in patients with bipolar mania [12]. Clinicians are asked to consider ADHD and its implications when considering disorders of circadian rhythm since these patients often exhibit a delayed chronotype and a preference for evening circadian rhythms [20].

The presence of psychotic features, which is sometimes observed in patients with BD experiencing a manic episode, is an example of a clear distinguishing characteristic that would not be seen in patients with even the most severe forms of ADHD [12].

Bipolar disorder is known for its episodic or oscillatory disease course, with clear and distinct manic and depressive that have remarkable variations in cycling. This is different from ADHD, in which the patients with hyperactive features exhibit these characteristics as stable traits rather than variable states seen in mood episodes of bipolar disorder [9].

Lastly, the onset of ADHD is earlier than typically observed for BD, with most symptoms first occurring before the age of 12. The age of onset of BD is typically later, with a peak in late adolescence and early adulthood [12].

### 3.3. ADHD and Borderline Personality Disorder

Much like bipolar disorder, the prevalence of ADHD in patients with BPD is high and has been described as an aggravating factor in patients who struggle with ADHD symptoms [21]. It is estimated that 27.2% of adult patients with ADHD meet diagnostic criteria for BPD, while roughly 18% have an established diagnosis [22]. It has also been observed that when ADHD symptomatology is more severe, the likelihood of being diagnosed with a personality disorder is increased [22]. These two disorders each have common risk factors, including adverse childhood experiences, difficult temperament, and family history—thus contributing to a great degree of the overlap observed in clinical practice [22].

One of the diagnostic criteria to consider when approaching the assessment of a patient who may have ADHD-BPD is the fact that the symptom of impulsivity can be associated with both diagnoses. Factors to consider, however, include the fact that these can be discernible based on the patterns of presentation. In ADHD, impulsivity is often represented via a pervasive pattern of impatience and interruption, characterized by talking over others and difficulty with waiting one’s turn. In BPD, the symptom domain of impulsivity is better observed in acts carried out while under the influence of intense emotion, including self-harm and disregard for safety [23]. The DSM-5 states that impulsivity in at least two areas must be present for diagnosis and that these must have the potential for damage to self. Examples of these impulsive behaviors include gambling, irresponsible spending of money, binge eating, substance abuse, unsafe sexual behaviors, and reckless driving—as well as behaviors or threats that are suicidal or self-mutilating in nature [11].

Emotional dysregulation is a feature of borderline personality disorder that is often represented by a series of thoughts, behaviors, and actions that are linked to an unstable sense of self—including frequent loss of temper, mood swings within the same day, and impaired stability in interpersonal relationships [11]. While emotional dysregulation is not a diagnostic criterion for ADHD, some recent findings have identified it as an important clinical feature in this population [24]. There is a great deal of anecdotal evidence demonstrating that adult ADHD patients experience symptoms of emotional irregularity, including frequent mood swings, impaired ability to tolerate stressful experiences, and labile moods—meaning that they are prone to quickly becoming angry, excited, or irritated. Up to 72% of patients in an ADHD sample reported experiencing these symptoms [25]. Of note, however, is the fact that the impulsive and emotional dysregulation features of ADHD are observed to be of lesser intensity and more likely to remit over time when compared to BPD, in which these traits are more pervasive and typically remain stable throughout adulthood [22]. The same patients who reported symptoms of emotional dysregulation also had stronger functional impairment, thereby further highlighting the importance of this feature [25]. While the evidence to support these findings is largely empirical, it allows us to appreciate the implications of ADHD-BPD comorbidity, as well as the complexity of navigating these diagnoses both when presenting separately and together.

One of the main differences between ADHD and BPD is the state of inner tension that is described by experts in the field of BPD; this is best described as underlying feelings of stress and instability that produce behaviors that often manifest as self-destructive. The state of inner tension is also said to drive the intense and unstable nature of BPD patient relationships, the insufficient sense of self, and dissociative or paranoid symptoms that are not observed in ADHD. Patients with BPD may experience paranoid thoughts, such as being hated by others, and they are often distressed by these thoughts [22].

There are important diagnostic considerations in patients with comorbid ADHD and BPD; both validated sources like the DSM-5, in addition to a surplus of anecdotal evidence from experienced clinicians, have identified a great deal of symptom overlap in each of these psychiatric conditions [23]. Discussions surrounding management approaches with an understanding of the rich complexity of patients with these disorders will help to increase the accurate diagnosis and appropriate treatment of their mental illnesses.

### 3.4. What Are the Main Distinguishing Features between ADHD and Borderline Personality Disorder?

The primary similarities between ADHD and BPD are in the symptom domains of impulsivity and emotional dysregulation. There are, however, subtle differences in each diagnosis that psychiatrists and psychiatric care providers should be aware of.

Impulsivity is defined differently by the DSM-5 in the context of each diagnosis. For BPD, the manual describes impulsive behaviors as those that are self-damaging, including impulsive sexual behaviors, spending, binge eating, or reckless driving. ADHD-related impulsivity is described as difficulties with waiting patiently, interrupting others, and blurting out answers before questions are completed [11]. Again, we examine closely the different types of impulsivities that are observed in ADHD—both cognitive impulsivity and motor impulsivity, which manifest in some of the above presentations [13]. This differs from the behavioral impulsivity that is observed in patients with BPD, as these impulses tend to be driven less by cognition and motor activity and more by intense emotion [23]. These two types of impulsivity are clearly distinct from one another and should be carefully dissected in the assessment process.

Experts in the field of psychiatric comorbidities have identified ADHD-related impulsivity to be largely trait-based impulsivity, meaning that the impulsive characteristics of one’s personality are static, unchanging, and present in all social situations and circumstances. In contrast, impulsivity in BPD is clearly more state-based, indicating that impulsive behaviors are elucidated when the patient is experiencing high levels of stress [23]. In one 2016 study, researchers identified that ADHD patients showed higher impulsivity rates (as measured with the self-reported Barratt Impulsivity Scale and UPPS Impulsive Behavior Scale) under normal conditions, indicating higher trait-based impulsivity, while patients with BPD showed equal levels of self-reported impulsivity and stronger impulse control deficits when compared to the ADHD and control groups after the induction of stress [26].

While emotional dysregulation is not a diagnostic criterion for ADHD like it is for BPD, researchers and clinicians alike have identified it as a finding in most ADHD patients. Emotional instability in BPD is a core psychopathological characteristic—the DSM-5 describes marked emotional reactivity, intense episodes of dysphoria and irritability, and uncontrollable anger [11]. Up to 70% of adults with ADHD may also experience emotional dysregulation as a persistent symptom [27]. The discerning feature between patients with BPD and patients with ADHD and emotional dysregulation is that those with ADHD appear to have better control over their emotions and are more likely to use cognitive strategies to temper their reactivity to stressful situations [23]. The inadequate cognitive regulation of emotions is observed in studies of BPD, in which patients persistently struggle with identifying their own emotions; they are also more likely to use maladaptive strategies such as rumination, thought suppression, and acceptance [28]. The reduced ability to control one’s impulses in BPD has been biologically linked to deficits in prefrontal emotional processing in one study of male patients with the disorder—this helps to explain why those with BPD are more prone to outbursts of anger and aggressive behavior, something that is not as typically seen in ADHD [29].

As discussed previously, self-harm in the context of BPD-related impulsivity is one of the hallmark features of this personality disorder; one previous study solemnly reported that up to 75% of patients with BPD had attempted suicide at least once [30]. The suicidal and self-harm behavior is attributable to both the impulsivity and emotional dysregulation domains of the BPD diagnosis [11]. The rates of suicidal behavior in ADHD are lower but still important, with roughly 10% of adult ADHD patients having a history of one or more suicide attempts. It is theorized, however, that suicidality in ADHD is related to the symptoms of low self-esteem and the increased prevalence of comorbidities and dysfunctional factors that accompany the disorder [31].

Interpersonal deficits are another key characteristic of BPD that, when seen in ADHD, would be considered more of an indirect effect rather than a core symptom [32]. Adults with ADHD are known to have some interpersonal difficulties; however, these are often more related to other factors. These patients are known to struggle in the workplace environment due to issues like absenteeism, tardiness, and error-prone behaviors; this consequently can predispose them to interpersonal conflict and relationship strain with employers and colleagues [33]. While relationships are frequently short-lived, emotionally intense, and unstable in those with BPD, this is largely associated with the emotional dysregulation and instability in the sense of self that is known to affect this population [11]. In addition to occupational factors, those with ADHD are more likely to have children with ADHD, which can contribute to significant parenting challenges and can give rise to disordered substance use—all of which are issues that can accentuate relationship difficulties that may occur in adults with ADHD without a personality disorder [33].

Disturbances of identity are a unique and interesting feature of a few select psychiatric disorders, among which include dissociative disorders and BPD. These symptoms are described as markedly unstable senses of self, often represented by a fragmented identity and sudden and dramatic shifts in entities like values, opinions, friends, sexual identity, and career aspirations [11]. Identity disturbances such as these are often associated with a childhood history of trauma, often in which individuals have been exposed to interpersonal abuse, neglect, conflict, or loss in the home—patients with BPD are as much as 13× more likely to report experiencing adversities such as these [34]. While trauma is not always a part of the BPD patient’s history, the strong correlation between adverse childhood experiences (ACEs) and symptoms of identity disturbances warrants significant consideration. Adults with ADHD may exhibit disorganized or novelty-seeking behaviors, but identity disturbances are unique to BPD and may manifest as patterns of behavior that are chaotic in nature, as well as hyper-investment in relationships and goals that lack coherence over time [35].

Identity disturbances may be connected to chronic feelings of emptiness, which is another DSM-5 criterion that is specific to borderline personality disorder [11].

One may argue that the low self-esteem that is characteristic of ADHD may contribute to feelings of emptiness as well; however, there is a unique distinguishing factor between this and what is observed in BPD. One analysis of the disorder across the lifespan found that feelings of emptiness, as well as somatic and depressive symptoms, tend to worsen as patients get older [36]. This is in sharp contrast with the low self-esteem seen in ADHD, which is more likely to improve with appropriate treatment interventions and changes in the environment (i.e., transitioning from school to work) [33,37].

The final feature of BPD that is not observed in ADHD is paranoid ideation, which is described as a transient experience in which patients may undergo frantic efforts to avoid abandonment in response to real or imagined stress [38]. This is often an ongoing struggle for those living with BPD, as they have a heightened response to the behavior of others that can contribute to paranoia that they may have about a person or situation, putting further strain on their interpersonal relationships [39]. While anxiety is common in adult ADHD, it is considered a comorbidity rather than a characteristic of the disorder, and the anxiety often seen in this population is described as generalized rather than paranoid, suspicious, or mistrustful in the case of patients with BPD [40].

## 4. Management Implications

### 4.1. Bipolar Disorder

The management of patients with comorbid ADHD and bipolar disorder is an under-researched topic; however, there have been some findings established as effective and worthy of consideration. The clinical impact in patients with ADHD and bipolar disorder is arguably increased because of the impairment in functioning that they experience because of their ADHD, in between the cycling episodes of depression and mania that are characteristic of bipolar disorder. Many studies have identified additional risks that patients with BD are subject to in the presence of a comorbid ADHD diagnosis, including:

- earlier age of onset of BD
- increased frequency of mood episodes (especially depressive episodes)
- increased risk and number of suicide attempts
- increased likelihood of substance use disorder
- poor response to mood-stabilizing medications [16].

The treatment goals in bipolar disorder typically revolve around achieving and maintaining mood stability, as well as preventing recurrent episodes of mania, hypomania, and depression [41]. This will often entail the short-term management of mood episodes when they occur and long-term maintenance of treatment plans to minimize recurrence. Medications remain the cornerstone of BD treatment, with the most commonly used medications being mood stabilizers and antipsychotics, used in conjunction with psychotherapy and lifestyle modifications when available [42]. Treatment for bipolar disorder, however, can be an arduous process as clinicians grapple with optimizing mood stabilization and minimizing adverse effects. Additional complicating factors include treatment non-adherence, challenging social circumstances, and unavoidable triggers for mood episodes [41]. It is crucial that physicians and other care providers who treat patients with BD use a patient- and family-centered approach and that they enlist multidisciplinary professionals when appropriate.

When BD and ADHD co-occur, among the first steps in developing a treatment plan is determining which condition and which symptoms are most severe and disabling. Typically, mood stabilization is a priority because untreated mood episodes in BD can have more devastating consequences, including hospitalization [12]. Once mood stabilization is achieved, the ADHD symptoms can begin to be addressed; another challenge arises here because it is known that some medications used to treat ADHD, particularly stimulants, can pose a risk for mood destabilization in individuals with BD [43]. A great deal of caution must be exercised when trying to treat BD and ADHD using monotherapy, and there is little data to support comprehensive treatment for both conditions using one drug. While some approaches will be discussed, it is important to be vigilant about the risk of manic and hypomanic induction when using stimulants and other medications in this patient population [44].

One 6-week prospective trial in 36 patients with diagnoses of ADHD and bipolar disorder showed that sustained-release bupropion was linked to a 55% reduction in ADHD symptoms and a 58% reduction in mood episodes associated with depressed mood in bipolar disorder patients, with little evidence of hypomania induction [45]. In this study, the patients were experiencing mild symptoms only on the Young Mania Rating Scale (YMRS) and Clinical Global Impression (CGI) scales, and there was a clinically significant reduction in mood symptoms at the end of the trial [45]. The positive response observed with bupropion helped to establish this medication as an effective treatment for ADHD-BD in the Canadian Network for Mood and Anxiety Treatments (CANMAT), with level 4 evidence [16]. The rate of medication-induced hypomania was low in this sample size [45].

Bipolar disorder, when not appropriately treated, may manifest itself in ways that may make it challenging for clinicians to discern between its symptoms and those of ADHD—thus, the first goal of treatment when approaching the management of these patients is to ensure that BD symptoms are adequately controlled, typically using mood-stabilizing treatments like lithium, lamotrigine, or divalproex sodium [12]. Methylphenidate (MPH) is a central nervous system (CNS) stimulant that has proven to be effective and tolerable for the treatment of ADHD in both children and adults [46]. While previous concerns surrounding treatment-emergent mania have been brought forward, a registry of over 2000 patients in Sweden demonstrated that this medication is a safer and more effective option in the treatment of ADHD in patients with BD who were receiving concomitant mood stabilizing therapy [44]. It is important to note that stimulants should not be used in patients who are experiencing active mania or psychosis and that those who are well-controlled should receive frequent monitoring [47]. This helps to further emphasize the importance of achieving euthymia before initiating stimulant therapy for comorbid ADHD in patients with bipolar disorder.

Other medications, such as modafinil and armodafinil, were significantly superior to placebo in some studies. One small study of 85 patients with BD demonstrated that modafinil use was associated with improved depressive symptoms in the study population [48]. Similarly, another trial using armodafinil demonstrated improved depressive symptoms in patients with type I bipolar disorder, and that it appears to be safer in terms of not increasing suicidality or manic symptoms; however, the rates of clinical response using armodafinil did not differ from placebo [48].

Atomoxetine is another commonly prescribed medication for the treatment of ADHD, which is a selective norepinephrine reuptake inhibitor—the first of the non-stimulant pharmacotherapy options [49]. While it has been investigated somewhat extensively as an option for pediatric and adolescent patients with ADHD-BD, the data are limited for the adult population. It is well-established that atomoxetine is associated with a statistically significant reduction in ADHD symptoms compared to placebo, but there have also been case reports of treatment-induced hypomania with this medication [43]. Atomoxetine can be considered as a potential option for patients whose bipolar symptoms are effectively managed, with close monitoring for the risk of hypomanic induction. The other ADHD pharmacotherapy to be considered for co-management is amphetamine salts, which are categorized as CNS stimulants. Recent research suggested that amphetamine-based products may carry double the risk than methylphenidate-based stimulants in inducing mania and psychosis [50].

Evidence has shown that there are noted interactions specifically between methylphenidate and anti-seizure medications, some of which may be used as mood stabilizers in the treatment of bipolar disorder [47]. The anti-seizure medications that are of special concern when used adjunctively with methylphenidate are phenytoin and carbamazepine; there are also a small number of case reports that describe a dyskinetic reaction when MPH is used alongside valproic acid [51]. The literature that is available for the treatment of comorbid ADHD and BD is limited; however, there are evidently various options in the realm of ADHD pharmacotherapy that have been associated with potential improvement in bipolar disorder symptomatology.

**Figure 1:**
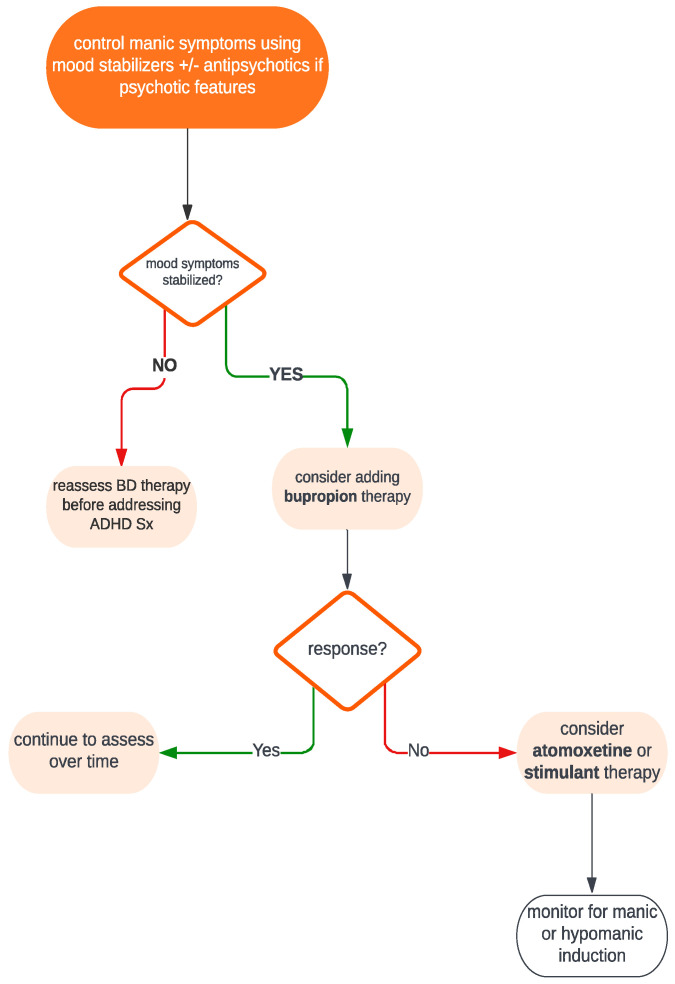
Simplified treatment algorithm for the pharmacological management of patients with comorbid ADHD and bipolar disorder.

It is best to think of the management of ADHD-BD using a hierarchical approach, with frequent assessment of treatment response, as seen in Figure 1.

While bupropion has shown some benefit in this population, it is important to consider the therapeutic advantages of using various mood stabilizers as well. Lithium, for example, which is well-established as effective BD pharmacotherapy, has demonstrated efficacy in reducing addictive and impulsive behaviors associated with ADHD and, therefore, may help to reduce complications [52]. One double-blind study found lithium to be comparable in effectiveness to methylphenidate in a predominantly male sample of ADHD patients [52].

There is limited data for using other mood stabilizers such as valproate and lamotrigine in the ADHD population; however, valproate is known to help alleviate symptoms such as emotional dysregulation and impulsivity, thereby warranting consideration for ADHD + BD co-occurrence [53]. Similarly, lamotrigine may have a positive impact on ADHD symptoms, especially when there is a depressive component to the clinical picture—this medication may be considered to help alleviate mood symptoms that contribute to impaired attention and concentration difficulties [54] (Hashimoto et al., 2021). The data for other mood stabilizers, including carbamazepine, are scant; studies of its use in BD have demonstrated a positive effect on the prevention of impulsivity, so it can be considered while further research is awaited [55].

The Canadian Network for Mood and Anxiety Treatments (CANMAT) has guidelines specifically for the treatment of bipolar disorder when it co-occurs with ADHD. Again, the stabilization of mood symptoms in BD using pharmacotherapy is identified as the priority before addressing ADHD symptoms [41]. ADHD pharmacotherapies were all tested for the management of ADHD symptoms in the adult BD population, and the evidence was varied. The medications atomoxetine, bupropion, and lisdexamfetamine are supported by level 4 evidence, while methylphenidate and mixed amphetamine salts are supported by level 3 evidence [41].

While atomoxetine, bupropion, and lisdexamfetamine are all supported by evidence of similar quality, because of limited data to support atomoxetine in the adult population as well as the risk of hypomanic induction, bupropion remains the preferred first-line agent for managing ADHD symptoms in the BD population. This is supported by recent literature that has demonstrated the clinically significant improvement in disease severity that bupropion has in patients with bipolar disorder, in addition to its dopaminergic properties that render it an effective choice for ADHD treatment [56]. There was a 2017 Cochrane review of a small number of studies that generally concluded that bupropion may lead to a small improvement in ADHD and may decrease ADHD symptoms [57]. Of importance, it is also considered not to pose a greater risk for phase switching (induction of hypomania or mania) when compared to any of the other antidepressants studied, including other SNRIs and SSRIs [56].

Key Points:

- Adequate mood stabilization in patients with ADHD-BD should be achieved using mood stabilizers prior to initiation of ADHD pharmacotherapy.
- There are some case reports of atomoxetine-associated hypomanic induction, while methylphenidate and amphetamine salts are considered to be low-risk medications in patients receiving appropriate mood stabilization therapy, but amphetamine salts carry double the risk than methylphenidate.
- Monitor for drug–drug interactions, particularly between methylphenidate and anti-seizure medications that are being used for mood stabilization.

### 4.2. Borderline Personality Disorder

Much like bipolar disorder, the management of ADHD is challenging for providers of psychiatric care. The diagnostic features of ADHD and borderline personality disorder (BPD) share overlapping characteristics, including impulsivity, emotional dysregulation, and issues with attention and concentration. There is also evidence of greater treatment resistance in patients with comorbid ADHD and BPD when compared to those with either disorder in isolation [23].

There have been case reports published detailing the successful use of methylphenidate for the treatment of comorbid ADHD and BPD, demonstrating that methylphenidate monotherapy is effective not only for ADHD symptom management but also for aggression and self-injurious behavior seen in BPD [22]. There are many features of BPD and ADHD that overlap, and it is estimated that up to 1/3 of patients with BPD have a comorbid diagnosis of ADHD [58]. Because of the similarities in symptoms, including impulsivity, emotionality, and cognitive dysfunction, the well-known ADHD treatment methylphenidate was investigated in patients with a dual BPD diagnosis. One study demonstrated that participants in the methylphenidate treatment group had performed significantly better on tasks of decision-making, which was attributed to the changes in dopaminergic transmission mediated by the drug [59]. It was also shown that methylphenidate treatment in patients with ADHD-BPD resulted in fewer mood episodes and decreased likelihood of expressing anger, the latter of which is a known feature of emotional lability in BPD [60]. In one study with ADHD patients, the non-stimulant ADHD medication atomoxetine was superior to placebo for the management of emotional symptoms such as irritability and emotional volatility; the fact that these are also features of BPD indicates that atomoxetine therapy may be a potential therapeutic choice for patients who suffer from comorbid ADHD and BPD [61].

Similar investigations have identified that mood stabilization should be achieved before initiating ADHD treatment and that methylphenidate and amphetamine salts should both be avoided in patients with pervasive cluster B personality disorders that have malingering features [43]. According to the American Psychiatric Association guidelines, mood stabilization can be achieved using both psychotherapeutic and pharmacologic modalities [62]. Medication selection depends largely on the presenting phenotype of the patient; patients with high levels of impulsivity or agitation may benefit from a second-generation antipsychotic such as aripiprazole or olanzapine [63]. Certain anticonvulsants like topiramate and lamotrigine have some limited or mixed evidence for mood stabilization in BPD patients with features of anxiety and anger [63].

When available, psychotherapeutic modalities should be engaged with these patients to maximize treatment outcomes and allow patients to benefit from a multi-pronged approach to care. Dialectical behavior therapy (DBT) is a psychotherapeutic method adapted from the traditional cognitive-behavioral therapy (CBT) that focuses on skill building for patients with some function-impairing features of BPD, including an unstable sense of self, relationship instability, emotional lability, and impulsivity that may manifest as self-destruction. It is currently the first-line and most evidence-based treatment for BPD [64]. It is administered by a trained professional and can be performed in either the individual or group setting. DBT is well-known to be effective in managing BPD; however, one study in predominantly female patients with ADHD-BPD demonstrated that its efficacy in enhancing mindfulness skills and reducing feelings of depression and hopelessness are also appropriate and useful in the management of ADHD symptoms [60]. While DBT and other psychotherapy modalities are helpful, there are several factors that need to be considered when incorporating these approaches into the treatment plans—including patient preference, the therapeutic relationship between patient and therapist, and the availability of high-quality services in the community [39]. It has also been identified that effective pharmacological management of ADHD in the BPD setting will help to produce enhanced functioning, decreased distress and inner tension, greater control over behavior, and potentially stronger engagement in psychotherapy in patients with both diagnoses [10].

Aside from DBT, there are other therapeutic options that may be available to patients if there are trained clinicians with the ability to provide these specialized services. Mentalization-based therapy and transference-focused therapy are examples of alternative psychotherapeutic modalities that have been proven to be helpful in the outpatient management of BPD, although they have not yet been studied in the ADHD-BPD population. Mentalization-based therapy can also be administered as either individual or group therapy, and it helps patients work through their impulses and develop strategies to think before reacting [65]. Because of its ability to aid in the control of impulsive behaviors, it has been theorized that its principles may be applied to patients with ADHD of the hyperactive-impulsive subtype. Transference-focused therapy is a psychoanalytic technique that has also proven to be effective as a therapeutic modality for patients with BPD; while it requires extensive training and is not commonly practiced, it may be another consideration for patients with BPD and comorbidities in areas where it is available [66].

Many off-label medications have been trialed for the management of ADHD when comorbid with BPD. Some of the symptoms of BPD, including feelings of emptiness, fear of abandonment, and identity disturbance, have been proven to be resistant to treatment with various agents, including olanzapine and quetiapine [10]. Clonidine, an alpha-2 receptor agonist, is known to be an effective adjunct treatment for ADHD; its efficacy is poorly understood but hypothesized to be helpful in the setting of ADHD management because of its actions on neurotransmitters like norepinephrine [67]. This medication has been shown to reduce inner tension and the urge to engage in self-harm behaviors, characteristics often observed in BPD. Thus, it can be considered a management strategy for patients who present with diagnoses of both ADHD and BPD [10].

Clinicians should also consider the risks and management difficulties that may accompany the treatment of a patient with ADHD-BPD. It is well-documented that patients with BPD have a propensity to use substances due in part to the symptoms of impulsivity that they experience and the experiences of inner tension and suicidality that often provoke the use of alcohol and drugs as a coping mechanism [68]. It is estimated that up to 78% of patients with BPD have a comorbid substance use disorder, so clinicians should take caution when prescribing psychoactive medications like stimulants, which have the potential for abuse, to patients with BPD [69].

It is evident that research into the management implications of ADHD-BPD is limited and that further investigations should be performed to better understand the unique needs of this patient population.

Key Points:

Methylphenidate treatment can be helpful not just in the management of ADHD symptoms but also in certain features of BPD, including impulsivity and executive dysfunction.

- Mood stabilization should be achieved before initiating stimulant therapy, and special caution should be taken in patients with suspected malingering behaviors.
- Dialectical behavior therapy remains the gold standard treatment for BPD, and it has shown to be effective in mitigating certain features of ADHD as well.

#### 4.2.1. Strengths

This narrative review offers a thorough examination of ADHD comorbidity management in adults with BD and BPD while incorporating evidence from a variety of sources. The practical implications of this review are that it will help provide clinicians and researchers with guidance about recommendations for the diagnosis and management of a traditionally challenging patient population.

#### 4.2.2. Limitations

One of the limitations that may be relevant in this review is the risk of selection bias, as the inclusion of studies depended on predefined criteria, potentially excluding some relevant research that was not incorporated. There was also a scarce amount of data available on certain topics, potentially impacting the quality of the recommendations. The quality of evidence was not assessed in this narrative review. The evidence also primarily stems from populations where Western medicine is practiced, so caution is needed when applying its findings in other cultural contexts. Selecting studies written in the English language only may have resulted in excluding other important studies.

#### 4.2.3. Recommendations for Future Research

Future research into the management of BD and BPD in adults with comorbid ADHD should address several key areas. Primarily, more extensive and well-designed studies are needed to explore treatment options in further detail, like bupropion, atomoxetine, and other non-stimulant pharmacotherapies. Researchers may want to specifically focus on understanding the precise mechanisms underlying the interplay between ADHD and these comorbidities, allowing readers to better understand the neurobiological pathways at play. Larger-scale studies would also be beneficial to help diversify the patient population and allow for greater generalizability. Investigations into novel approaches, such as combination pharmacotherapies or other specific psychotherapeutic modalities, could be addressed. Because of the literature gap, the future of research in this field holds promise for better understanding the intricacies of these disorders and better serving the patient population.

In conclusion, the management of ADHD comorbidities in the practice of psychiatry remains a complex process that requires a multifaceted approach. Adequate mood stabilization using mood stabilizers should take precedence before initiating ADHD pharmacotherapy in patients with both BD and BPD. While methylphenidate and mixed amphetamine salts are considered relatively low-risk medications when administered in conjunction with mood stabilization therapy, careful monitoring for drug–drug interactions, particularly in the case of methylphenidate and anti-seizure medications, is essential to ensure the safety of the patient. It is important to highlight the apparent efficacy that some medications used to treat ADHD, including methylphenidate, atomoxetine, and bupropion, have for treating certain features of BD as well, like impulsivity and executive dysfunction. Moreover, DBT remains the gold standard for the treatment of BPD, and it has also shown effectiveness in mitigating some aspects of ADHD. In summary, a tailored and holistic treatment approach that prioritizes mood destabilization in those with BD and BPD and considers individual symptom needs when managing the co-occurring ADHD. Bupropion, while ongoing research is required to obtain further evidence, can be a valuable add-on therapy to stabilize BD or BPD patients for therapeutic support for both ADHD and depressive mood symptoms.

## Figures and Tables

**Table 1:** The distinguishing clinical features between symptoms of ADHD and symptoms of bipolar I disorder and bipolar II disorder.

	ADHD	Bipolar Disorder (Mania)
Onset	Childhood	Early adulthood (typically)
Course of illness	Stable	Episodic
Affective symptoms	Low self-esteem	Euphoria, irritability, grandiosity
Distractibility	Difficulty focusing	Thought acceleration and flight of ideas
Clinical presentation	Restless, hyperactive, acts as if ‘driven by motor’	Psychomotor agitation, pressured speech
Sleep	Sleep disturbance is not part of the DSM-5 criteria	Decreased need for sleep
Psychotic symptoms	Not present	May be present

## Data Availability

References are cited at the end of the article.
